# Data on combination of parabolic solar system with CH_4_ cycle and power tower solar system with Cu–Cl cycle for hydrogen production in the city of Ghardaia (Algeria)

**DOI:** 10.1016/j.dib.2018.10.110

**Published:** 2018-10-27

**Authors:** Chawki Ameur menad, Rabah Gomri, Djemoui Lalmi

**Affiliations:** a Climatic Department, University Mentouri Constantine 1, 25000 Constantine, Algeria; b Research Unit in Applied Renewable Energy (URAER-CDER), BP:88, Gart Team ZI, 47133 Bounoura, Ghardaia, Algeria

## Abstract

This data show the combination of parabolic through solar system with CH_4_ cycle and power tower solar system with Cu–Cl cycle for hydrogen production capacity in the city of Gharadaia which is located in the south of Algeria. A proper measurement of meteorological factors such as temperature, humidity, and solar irradiation has been done in the city of Ghardaia due to the solar concentration in this city. In the meantime thermo-chemical systems (Cu–Cl, CH4 cycles) have been integrated with the thermal solar systems through.

**Specifications table**

**Table:** 

Subject area	Physics
More specific subject area	Solar energy, Hydrogen production
Type of data	Table, graph, figure
How data were acquired	Parabolic through solar system with CH_4_ cycle, power tower solar system with Cu–Cl cycle
Data format	Filtered, analyzed,
Experimental factors	Measuring Potential of necessary climatic factors in the city of Ghardaia
Experimental features	Using the meteorological station for measuring the temperature, humidity, and solar irradiation in the city of Ghardaia
Data source location	The city of Gharadaia in Algeria
Data accessibility	Data are with this article
Related research article	[1]https://doi.org/10.1016/j.applthermaleng.2015.08.074
	[2]https://doi.org/10.1016/j.applthermaleng.2016.11.201

**Value of the data**

• This data can be used as reference for hydrogen production through thermochemical systems under Algerian climate.
• This data can be used to cover the energy demands in Algeria through hydrogen production.
• This data can be used in describing the measurement of the climate key factors in the city of Ghardaia which is considered as one of the reference points for hydrogen production from solar energy.
• This data can be used in comparing the energy efficiency of parabolic through the solar system and the power tower solar system.
• This data can be used in comparing the CH_4_ thermo-chemical cycle, and Cu–Cl thermo-chemical cycle.

## 1 Data

**Fig. 1:**
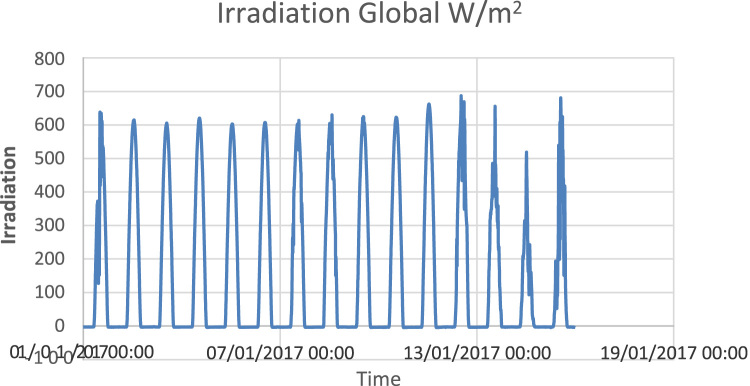
Solar irradiation in the city of Ghardaia (Algeria).

**Fig. 2:**
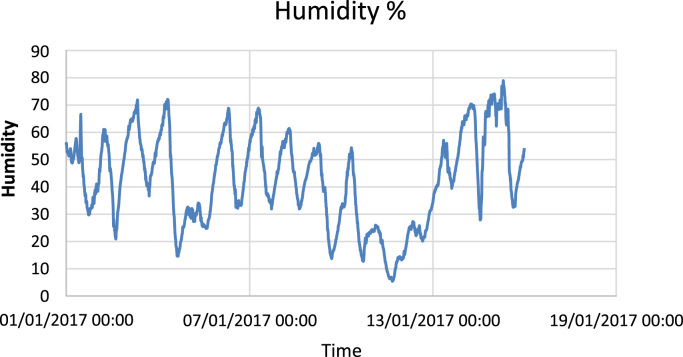
Humidity variation in the city of Ghardaia (Algeria).

**Fig. 3:**
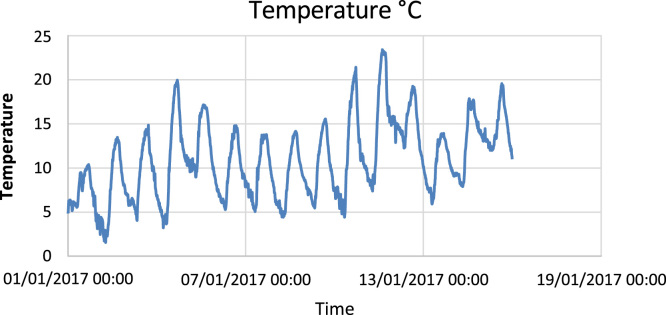
Temperature variation in the city of Ghardaia (Algeria).

The available data (Fig. 1, Fig. 2, Fig. 3) describe and show the solar irradiation, the humidity and the temperature in the city of Ghardaia in January 2017 where the solar irradiation is the dominant factor. It is clear that the solar irradiation is stable comparing to the other factors where the variation is instable during the same day. From Fig. 1, the solar irradiation varies between 0 W/m^2^ and 700 W/m^2^. Fig. 2 shows that the maximum humidity in January 2017 is 80%; in addition, the temperature varies between 2 °C and 23 °C (Fig. 3).

**Fig. 4:**
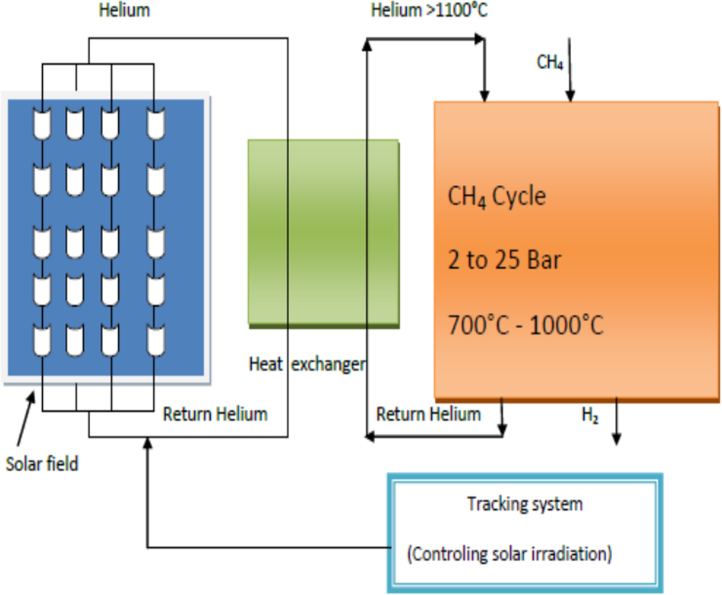
CH_4_ cycle through parabolic trough solar station.

**Fig. 5:**
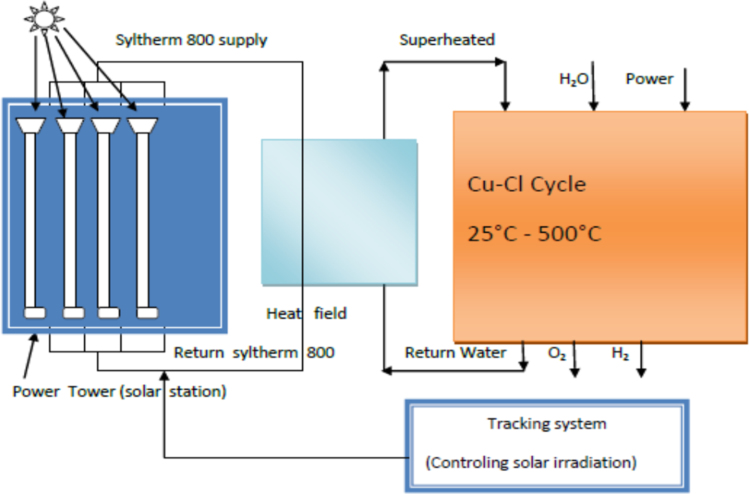
Cu–Cl cycle through the power tower solar system.

Fig. 4 presents the CH_4_ cycle through parabolic trough and Fig. 5 presents the Cu–Cl cycle through the power tower solar system.

From the calculation of heat loss in power tower solar system, Table 1 shows the energy efficiency and the super-heater outlet steam temperature.

**Table 1:** Energy efficiency of the power tower solar system for hydrogen production [1].

Time	Spring equinox 12:00 am
DNI (W/m^2^)	914
Solar field efficiency (%)	72.17
Evaporator specific mass flow rate (kg/m^2^ s)	540–880
Super-heater outlet flow rate (kg/s)	17.3
Super-heater outlet steam temperature (°C)	515 °C
Boiling receiver thermal efficiency (%)	88.16
Convective loss (%)	2.46
Radiative loss (%)	4.38
Reflective loss (%)	5
Super heater receiver thermal efficiency (%)	82.64
Convective loss (%)	4.84
Radiative loss (%)	9.52
Reflective loss (%)	3
Total receiver thermal efficiency (%)	86.55%

## 2 Calculation of energy efficiency of the solar parabolic trough collector system

**Fig. 6:**
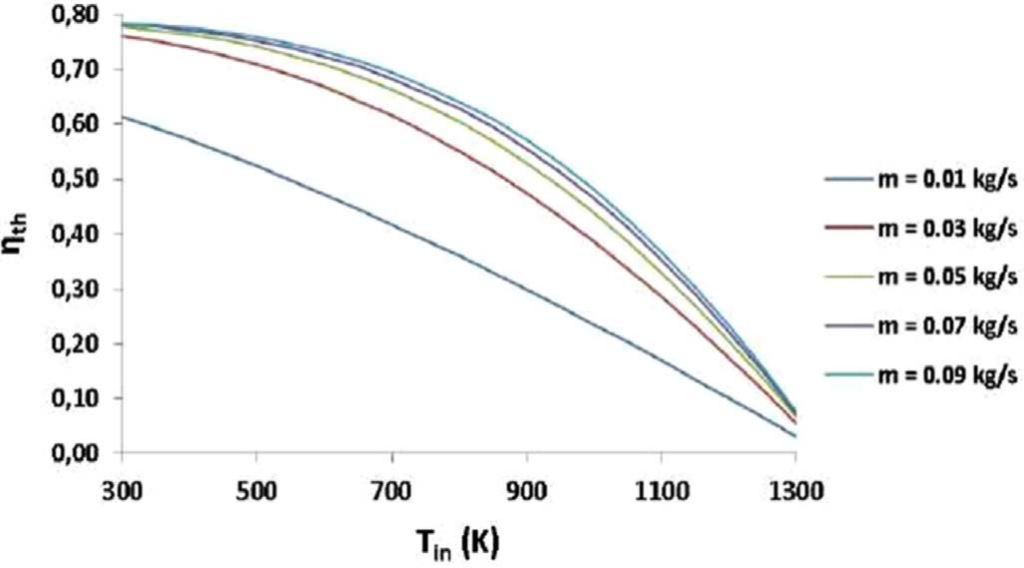
Thermal efficiency of helium for various mass flow rates [2].

**Fig. 7:**
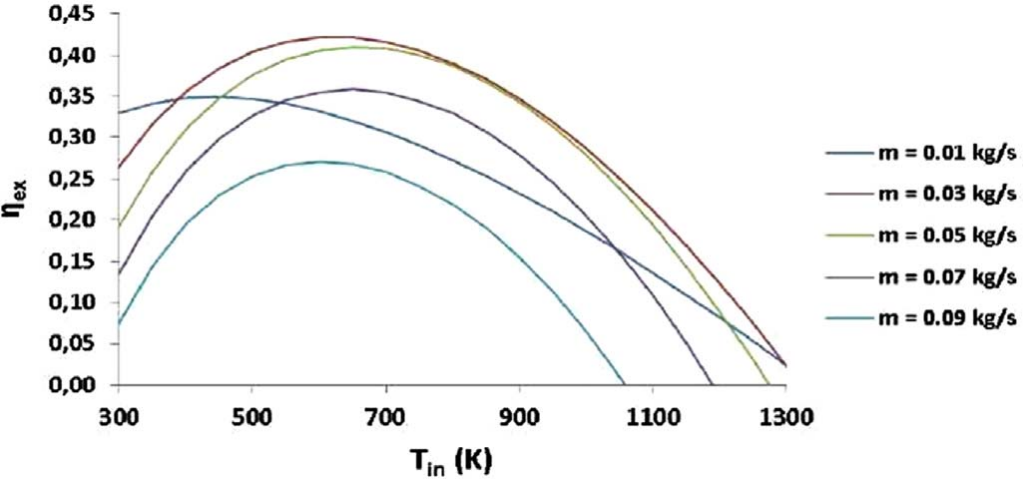
Exergetic efficiency of helium for various mass flow rates [2].

The calculation is based on comparison between thermal and exergetic efficiency of the Solar Parabolic trough collector system for hydrogen production which has been developed by [2], and has been described in (Fig. 6 and 7).

From both previous calculations, it is clear that there is a possibility for hydrogen production from CH_4_ and Cu Cl cycles through thermal solar energy systems in the city of Ghardaia. Table 2 shows the suitable thermal solar energy system to the right thermo-chemical cycle.

**Table 2:** The suitable thermal solar energy system to the right thermo-chemical cycle.

Thermal solar technology for hydrogen production	Energy efficiency	Temperature
Solar parabolic trough collector system [2].	42.21%	>826.85
Power tower [1].	86.55%	515 °C

The calculation of selected cycles to produce hydrogen is based on data given in Table 3.

**Table 3:** Mathematical calculation of hydrogen production from CH_4_ cycle and Cu–Cl cycle.

Cycle	City	Solar irradiation (W/m^2^)	The efficiency of solar system %	Hydrogen productivity (MJ/kg H_2_)
CH_4_ cycle (Parabolic trough collector)	Ghardaia	Measured	42.21% (>826.85 °C)	165 MJ/kg H_2_[10]
Cu–Cl cycle (Power tower)	Ghardaia	Measured	86.55% (515 °C)	195.7 MJ/kg H_2_[12]

## 3 Experimental, design, materials, and methodes

**Fig. 8:**
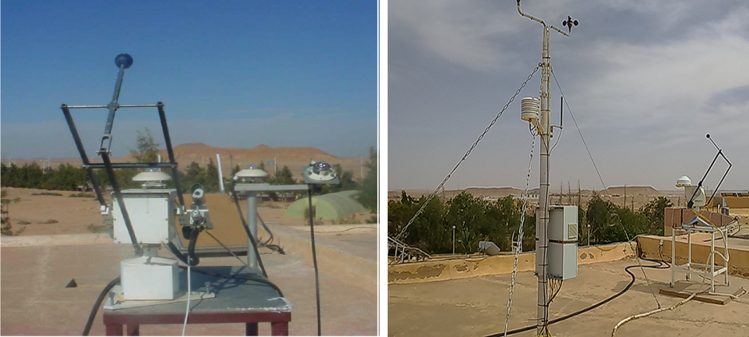
Meteorological power station in the city of Gharadaia (URAE: Research Unit in Applied Renewable Energy in the city of Gharadaia).

Measuring potential of necessary climatic factors in the city of Ghardaia: These data have been measured in the meteorological power station in the city of Ghardaia in January 2017 (Fig. 8) where the necessary climatic factors are temperature, humidity, and solar irradiation have been explained the ability of hydrogen production capacity through CH_4_ and Cu Cl cycles under the Algerian climates where the city of Ghardaia is considered the reference. The measurements have been taken in every 10 min. The experience is based on:

• measuring the solar irradiation in the city of Ghardaia;
• motivated the solar irradiation by the efficiency of the selected solar system;
• compare the energy obtained with the productivity of hydrogen (the necessary power to produce 1 kg of H_2_) from each cycle.

Comparing to the other thermo-chemical cycle, Cu-Cl cycle is the suitable solution on cloudy days due its relatively low temperature requirement [3]. Extensive research on pathways to find potential hydrogen demand has been developed, especially focusing on suitable methodologies to produce hydrogen from combination thermal solar energy systems, with thermo-chemical cycles. One of these researches has been developed about Cu-Cl cycle, Kalina cycle, and electrolyser for hydrogen production [4]. Another paper, has been developed about the integration of the receiver- reactor, with the energy collected in Cu-Cl cycle for hydrogen production [5]. Coupling between solar parabolic trough collector system, with CH_4_ cycle is considered to be one of the most important ways for hydrogen production. For this reason many researchers have involved in this pathway to increase hydrogen production. Stéphane Abanades, Gilles Flamant [6] have studied Solar hydrogen production from the thermal splitting of methane in a high temperature solar chemical reactor. The obtained results, CH_4_ mole fraction has a strong effect on the final chemical conversion of methane. Sylvain Rodat et al [7] have studied Hydrogen production from solar thermal dissociation of natural gas: development of a 10 kW solar chemical reactor prototype. Experimental results explain that methane conversion and hydrogen yield of up to 98% and 90%, respectively. Stéphane Abanades Gilles Flamant [8] have developed an experience for studying and modeling of a high- temperature solar chemical reactor for hydrogen production from methane cracking. The obtained results showed that the conversion of CH_4_ and yield of H_2_ can exceed 97% and 90%, respectively.

The obtained data have given a clear idea to researchers about hydrogen production to cover energy demands under Algerian climate.

**Fig. 9:**
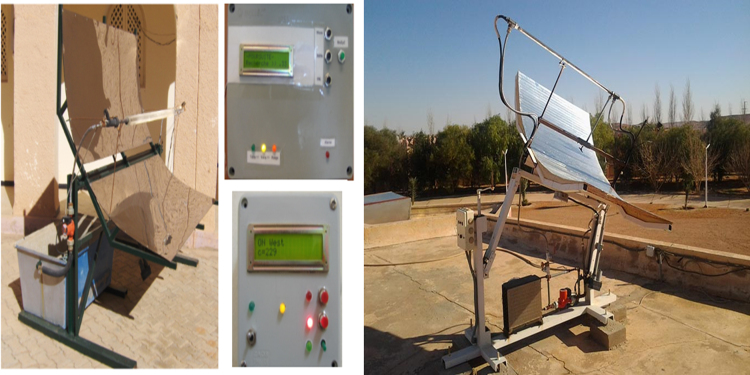
Solar parabolic trough collector system in the city of Gharadaia (URAER: Research Unit in Applied Renewable Energy in the city of Gharadaia).

**Fig. 10:**
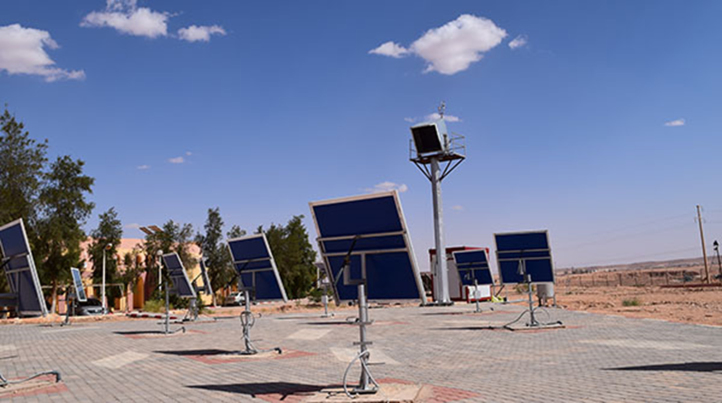
Power tower solar system in the city of Gharadaia (URAER: Research Unit in Applied Renewable Energy in the city of Ghardaia).

The design of the best solar system in the city of Ghardaia to exploit the existing data that have been taken under consideration under different climatic factors. Figs. 9 and 10 show the suitable design for combining the existing data and hydrogen production.

## 4 Hydrogen production from solar energy and thermo-chemical cycles as a future solution

**Fig. 11:**
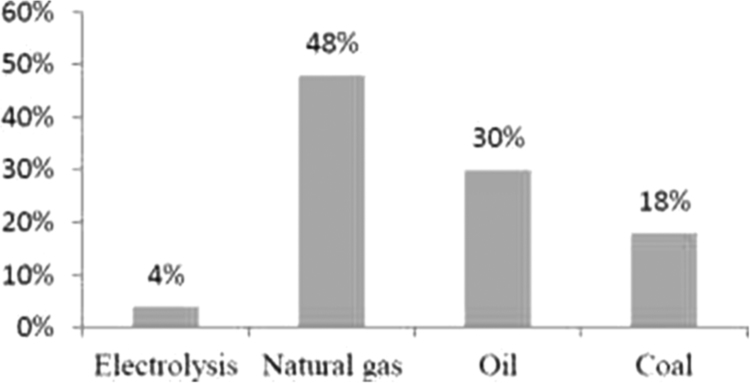
Origin of hydrogen currently produced worldwide [9].

Fig. 11 shows the hydrogen produced in the world [9].

Table 4 shows the hydrogen productivity from CH_4_ cycle and Cu–Cl cycle.

**Table 4:** Hydrogen productivity from solar thermal energy and thermo-chemical cycle.

Hydrogen cycle production	Productivity of hydrogen MJ/kg H_2_	Thermal solar system
CH_4_ (700–1000°C) [10]	165 MJ/kg H_2_[10]	Solar parabolic trough collector system
Cu–Cl (25–500°C) [11]	195.7 MJ/kg H_2_[12]	Power tower solar system

**Fig. 12:**
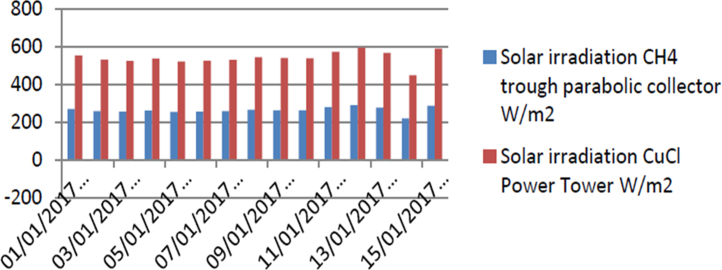
Solar irradiation obtained from thermal solar systems to hydrogen in the city of Ghardaia.

Fig. 12 gives the comparison between solar irradiation absorbed by a trough parabolic collector system in CH_4_ cycle and the power tower solar system in Cu–Cl cycle. The energy efficiency in the thermal solar system integrated with Cu–Cl cycle (86.55%) is higher than the energy efficiency in the thermal solar system integrated in CH_4_ cycle (42.21%).

**Fig. 13:**
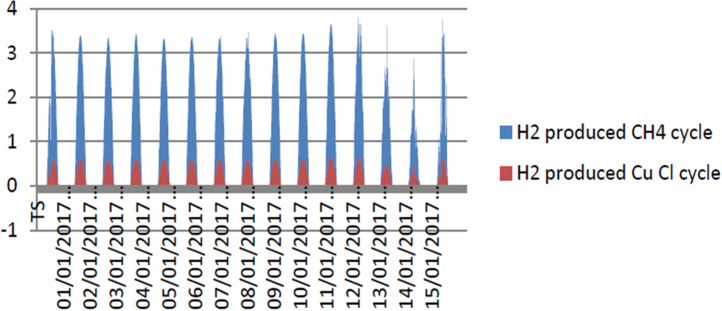
Hydrogen produced from CH_4_ cycle and Cu–Cl cycle.

Fig. 13 explains that the amount of hydrogen produced from CH_4_ cycle integrated with the trough parabolic collector system (energy efficiency = 42.21%) is higher than the amount of hydrogen produced in Cu–Cl integrated with the power tower system (energy efficiency = 86.55%).

**Fig. 14:**
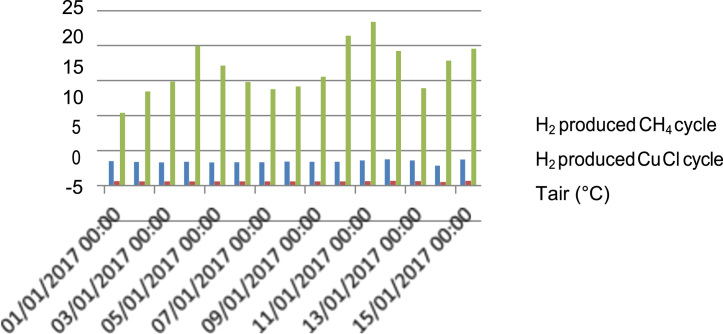
Hydrogen production from CH_4_ cycle and Cu–Cl cycle in function of air temperature.

Fig. 14 describes the variation of temperature on function of hydrogen production from CH_4_ cycle and Cu–Cl cycle. From the obtained results, the temperature has a strong impact on hydrogen production under Algerian climate through CH_4_ cycle and Cu–Cl cycle. In addition, the amount of hydrogen produced from CH_4_ cycle is much better than Cu–Cl cycle.

**Fig. 15:**
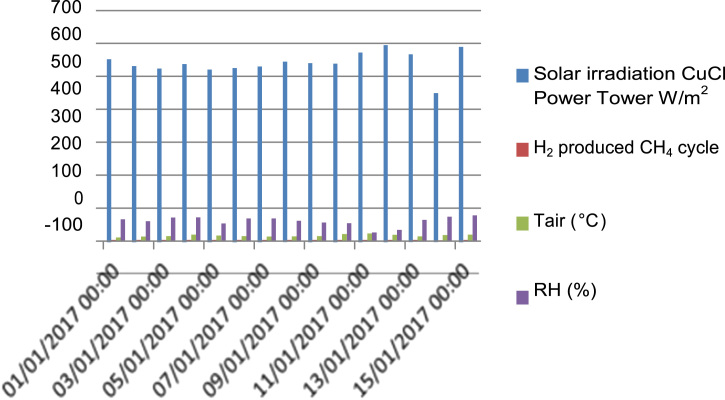
Hydrogen production from CH_4_ cycle and Cu–Cl cycle on function of climatic factors in the city of Gharadaia.

Fig. 15 shows the hydrogen production from CH_4_ cycle and Cu–Cl cycle on function of climatic factors in the city of Gharadaia. The humidity does not affect the hydrogen production from CH_4_ cycle, and Cu–Cl cycle due to strong variation of temperature, and the solar irradiation in the city of Ghardaia.
